# Therapeutic Potential of Polar and Non-Polar Extracts of *Cyanthillium cinereum In Vitro*

**DOI:** 10.1093/ecam/nep155

**Published:** 2011-06-23

**Authors:** Gunjan Guha, V. Rajkumar, R. Ashok Kumar, Lazar Mathew

**Affiliations:** School of Biotechnology, Chemical and Biomedical Engineering, VIT University, Vellore 632 014, Tamil Nadu, India

## Abstract

*Cyanthillium cinereum* (Less.) H. Rob. (Asteraceae) has been traditionally known for its medicinal properties, all aspects of which are yet to be exploited. This study was aimed at investigating the therapeutic potential of polar (methanolic and aqueous) and nonpolar (hexane and chloroform) crude extracts of the whole plant. Several parameters including free-radical (DPPH^•^, ABTS^•+^, H_2_O_2_ and ^•^OH) scavenging, reducing power, protection of DNA against oxidative damage, cytotoxicity, inhibition of oxidative hemolysis in erythrocytes, total phenolic content and inhibition of lipid peroxidation were examined. All the free-radical generating assay models demonstrated positive scavenging efficiency with differential but considerable magnitudes for the four extracts. However, only the hexane extract showed significant H_2_O_2_ scavenging effect. Lipid peroxidation was estimated by thiobarbituric acid-malondialdehyde (MDA) reaction, and a high degree of inhibition was shown by all the extracts. Reducing power of the polar extracts was higher than the non-polar ones. All extracts showed a concentration-dependent increase in phenolic contents. Oxidative damage to erythrocytes was hindered by all extracts in diverse degrees. XTT assay showed that all extracts have mild cytotoxic property. The aqueous extract evidently demonstrated protective effect on pBR322 plasmid DNA against oxidative breakdown. These results suggested the potential of *C. cinereum* as medicine against free-radical-associated oxidative damage and related degenerative diseases involving metabolic stress, genotoxicity and cytotoxicity.

## 1. Introduction

Atoms or molecules containing one or more unpaired electrons are termed as free radicals which are accountable for tissue degeneration by means of DNA and protein damage and lipid peroxidation. Oxidative stress associated with free radicals is involved in the pathophysiology of aging and various age-related ailments such as cataracts, atherosclerosis, diabetes, Alzheimer's disease, and so forth. The extent of damage caused by free radicals might be mitigated through supplementation with one or more antioxidants [1]. Various compounds with differential antioxidant properties are found in floral resources which are considered to have high potential in the context of therapeutic approaches to encounter and prevent free radical damage. Diverse medicinal plants have been screened and assessed for properties in antagonism to free-radical-induced oxidative stress [2].

*Cyanthillium cinereum* (Less.) H. Rob. (synonym: *Vernonia cinerea* (Linn.) Less.), commonly known as little ironweed, is a common annual weed (Asteraceae) with a wide range of geographical distribution. The plant has great medicinal value in diverse traditional usage in different nations, and also gets recognition in the *Ayurvedas* [3]. The whole plant is used in decoction or infusion to treat fever [4]. It provides remedy for spasms of the urinary bladder and strangury, and is often combined with quinine to treat malaria [3]. Sesquiterpene lactones, which possess antimalarial activity, have been isolated from the plant [5]. *Cyanthillium cinereum* has therapeutic potentials against asthma [6], cancer [7], cholera, colic pain, cough, diarrhea, dysentery, impotency and night-blindness [4]. The seeds are used as a source of alexipharmic and anthelmintic drugs, and as an alterative in leprosy and chronic skin diseases [3]. *Cyanthillium cinereum* leaves have analgesic, antipyretic and anti-inflammatory effects [8]. Paste of stem/bark is used to heal cuts, while flowers are traditionally used to treat conjunctivitis [3], arthritis [9] and rheumatism [10]. Root infusion is used as an antidote to scorpion sting and snake venom [3].

The present study is aimed at estimating the diverse therapeutic potentials of non-polar (hexane and chloroform) and polar (methanolic and aqueous) extracts of *C. cinereum* (whole plant) *in vitro* with respect to antioxidant and free-radical-scavenging properties, inhibition of lipid peroxidation, cytotoxicity and protection from DNA and cell damage.

## 2. Methods

### 2.1. Chemicals and Other Reagents

1,1-Diphenyl-2-picrylhydrazyl (DPPH), thiobarbituric acid (TBA), ethylene diamine tetraacetic acid (EDTA), gallic acid, ascorbic acid, trichloroacetic acid (TCA), phenazine methosulfate (PMS) (also known as N-methylphenazonium methosulfate), dimethyl sulfoxide (DMSO), Dulbecco's phosphate buffered saline (PBS) (Ca^2+^/Mg^2+^ free) and L-15 (Leibovitz) cell culture medium (with l-glutamine) were purchased from Himedia Laboratories Pvt. Ltd. (India). 2,2′-Azinobis-3-ethylbenzothiazoline-6-sulfonic acid (ABTS) and Trolox (6-hydroxy-2,5,7,8-tetramethyl chroman-2-carboxylic acid) were purchased from Sigma Aldrich Chemical Co. (Milwaukee, WI, USA). XTT {2,3-*bis*(2-methoxy-4-nitro-5-sulfophenyl)-5-[(phenylamino)carbonyl]-2H-tetrazolium hydroxide} was obtained from Sigma Chemical Co. (St. Louis, MO, USA). Folin-Ciocalteau reagent was procured from Sisco Research Lab (India). The remaining chemicals and solvents used were of standard analytical and HPLC grades, respectively. pBR322 was purchased from Medox Biotech India Pvt. Ltd (India). MDA-MB-435S (human breast carcinoma) cell line was obtained from National Centre for Cell Science (Pune, India).

### 2.2. Plant Material

*Cyanthillium cinereum* (whole plant) was collected in the month of July 2007 from Vellore district (12°55′N, 79°11′E), Tamil Nadu, India, and identified at Botanical Survey of India, Southern Circle, Coimbatore, India (BSI/SC/5/07-08/Tech.-523, 13 July 2007). Healthy plants were screened and thoroughly washed. The cleansed plants were freeze-dried for 2 months at –80°C in a MDF-U32V V.I.P.^TM^ Series 86°C UltraLow Temperature Freezer (Sanyo Biomedical, IL, USA). The dried plants were powdered for the preparation of extracts.

### 2.3. Preparation of Extracts

Fifty grams of whole plant powder was serially extracted with hexane, chloroform, methanol and water using Soxhlet apparatus. These crude extracts were concentrated at 40°C under reduced pressure (hexane: 360 mbar; chloroform: 474 mbar; methanolic: 337 mbar; aqueous: 72 mbar) with Rotavapor R-215 (BÜCHI Labortechnik AG, Switzerland) to yield dry extracts. The final quantities of the hexane, chloroform, methanolic and aqueous extracts obtained were 2.22, 0.9, 9.79 and 5.21 g, respectively.

### 2.4. DPPH Radical Scavenging Activity

The DPPH assay was done according to the method of Brand-Williams et al. [11] with some modifications. Two milliliters of extract solution (10, 20, 40, 60, 80 and 100 *μ*g ml^−1^) made in methanol was added to 1 ml of DPPH^•^ solution (0.2 mM ml^−1^ methanol) and mixed vigorously. The mixture was incubated in dark at 20°C for 40 min. Absorbance was measured at 517 nm using a Cary 50 UV-Vis spectrophotometer (Varian, Inc., CA, USA) with methanol as blank. Trolox was used as positive control.

The level of percentage inhibition of DPPH^•^ by the different extracts was calculated according to the following formula:

(1) $$\text{\% Radical}\;\;\text{scavenging}=\left[\frac{A_{C}-A}{A_{C}}\right]\times 100,$$

where *A*_C_ is the absorbance of the control and *A* is the absorbance of sample. Percentage scavenging was also evaluated in Trolox equivalence.

### 2.5. ABTS Radical Scavenging Activity

ABTS assay was performed according to the protocol of Arnao et al. [12]. The stock solution was prepared by mixing equal volumes of 7.4 mM ABTS^•+^ solution and 2.6 mM potassium persulfate solution followed by incubation for 12 h at room temperature in dark. Working solution was prepared freshly before each assay by diluting the stock solution by mixing 60 ml methanol to 1 ml stock solution. Different concentrations of the phyto-extracts were taken in test tubes. Three milliliters of ABTS^•+^ working solution was added to each tube and allowed to react for 2 h in dark. Absorbance was taken at 734 nm. Percentage radical scavenging activity was calculated using a similar formula as employed for the DPPH assay, and plotted along with Trolox equivalence.

### 2.6. Lipid Peroxidation Inhibition Efficiency

Inhibition efficiency of lipid peroxidation inhibition (LPI) was estimated by TBA assay [13]. A 6-week-old female Wistar albino rat weighing approximately 150 g was sacrificed under general ethereal anesthesia and its liver was excised. In total, 10% (w/v) liver homogenate was prepared in Dulbecco's PBS (Ca^2+^/Mg^2+^ free) (pH 7.4), and centrifuged at 3000 rpm (503 × g) for 15 min to obtain a clear supernatant. Diverse concentrations of each extract were taken in different test tubes and evaporated to dryness at 80°C. One milliliter of 0.15 M potassium chloride and 0.5 ml of the obtained supernatant were added to each tube. Lipid peroxidation was initiated by the addition of 100 *μ*l of 0.2 mM ferric chloride and incubated at 37°C for 30 min. Two milliliters of ice-cold 0.25 N-hydrochloric acid containing 15% TCA and 0.38% TBA was added to stop the peroxidation reaction, followed by an incubation of 1 h at 80°C. The samples were brought down to room temperature and centrifuged at 7500 rpm (3144 ×g) for 15 min. Absorbance of the supernatant was measured at 532 nm.

Percentage LPI was calculated by the following formula:

(2) $$\text{\% LPI}=\left[\frac{A_{C}-A}{A_{C}}\right]\times 100,$$

where *A*_C_ is the absorbance of the control and *A* is the absorbance of the extract sample. LPI of the extracts were compared with that of butylated hydroxy toluene (BHT) and expressed in BHT equivalence.

This experiment was performed according to the guidelines of the “European Convention for the Protection of Vertebrate Animals used for Experimental and Other Scientific Purposes” (and its appendix) with the approval of the institutional animal ethical committee (PSGIMSR/27.02.2008).

### 2.7. Total Phenolic Content Estimation

The total phenolic content of the different extracts of *C. cinereum* was determined using the Folin-Ciocalteau reagent method [14]. To 50 *μ*l of each extract, 2.5 ml of Folin-Ciocalteau reagent (1/10 dilution) and 2 ml of 7.5% Na_2_CO_3_ (w/v) were added and mixed well. The blend was incubated at 45°C for 15 min. The absorbances of all samples were measured at 765 nm with Na_2_CO_3_ solution (2 ml of 7.5% Na_2_CO_3_ in 2.55 ml of distilled water) as blank. The results were expressed as gallic acid equivalence (GAE) in micrograms.

### 2.8. H_2_O_2_ Scavenging Activity

H_2_O_2_ scavenging activities of the four extracts were determined by the method of Ruch et al. [15]. Extracts were prepared in Dulbecco's PBS (Ca^+2^/Mg^+2^ free) in concentrations 12.5, 25, 50, 100 and 250 *μ*g ml^−1^. Two milliliters of each extract solution was separately added to 1.2 ml of 4 mM H_2_O_2_ solution (prepared in PBS), and incubated for 10 min at room temperature. The absorbance of each of the solutions was measured at 230 nm with PBS as blank.

Percentage H_2_O_2_ scavenging activity (HPSA) was calculated using the following formula:

(3) $$\text{\% HPSA}=\left[\frac{C_{A}-S_{A}}{C_{A}}\right]\times 100,$$

where *C*_A_ is the absorbance of control and *S*_A_ is the absorbance of individual extract samples.

### 2.9. Hydroxyl Radical Scavenging Activity

^•^OH radical scavenging activity (HRSA) of the extracts was estimated by the method of Klein et al. [16]. Extracts were taken in different amounts (50, 100, 150, 200 and 250 *μ*g) in test tubes. One milliliter of iron-EDTA solution (0.13% ferrous ammonium sulfate and 0.26% EDTA), 0.5 ml of 0.018% EDTA and 1 ml of 0.85% (v/v) DMSO (in 0.1 M phosphate buffer, pH 7.4) were added to the test tubes, followed by 0.5 ml of 0.22% (w/v) ascorbic acid. The tubes were capped tightly and incubated on a water bath at 85°C for 15 min. Post incubation, the test tubes were uncapped and 1 ml of ice-cold TCA (17.5% w/v) was added in each immediately. Three milliliters of Nash reagent (7.5 g of ammonium acetate, 300 *μ*l glacial acetic acid and 200 *μ*l acetyl acetone were mixed and made up to 100 ml with distilled water) was added to all the tubes and incubated at room temperature for 15 min. Absorbance was measured at 412 nm.

Percentage HRSA was calculated by the following formula:

(4) $$\text{\% HPSA}=\left[\frac{A_{0}-A_{S}}{A_{0}}\right]\times 100,$$

where *A*_0_ is absorbance of the control and *A*_S_ is that of the individual samples.

### 2.10. Reducing Power Estimation

Reducing power of the four extracts was estimated by the method of Yildirim et al. [17]. Twenty microliters of each extract was mixed with 2.5 ml of 0.2 M phosphate buffer (pH 6.6) and 2.5 ml of 1% potassium ferricyanide. The reaction mixture was incubated at 50°C for 30 min. In total, 2.5 ml of 10% TCA was then added to the mixture and centrifuged for 10 min at 3000 rpm (503 × g). In total, 2.5 ml of the uppermost layer of the supernatant was collected and mixed with 2.5 ml of distilled water and 0.5 ml of 0.1% ferric chloride. Absorbance was measured at 700 nm. Increase in absorbance of the reaction mixture indicated increased reducing power.

### 2.11. DNA Damage Inhibition Efficiency

Efficiency of the extracts as potential DNA protectors was tested by treating pBR322 plasmid DNA with H_2_O_2_, which was simultaneously photolysed by UV (8000 *μ*W cm^−2^) [18]. In total, 1 *μ*l aliquots of pBR322 (200 *μ*g ml^−1^) were taken in four microcentrifuge tubes. Fifty micrograms of each of the four extracts was separately added to the tubes. Four microliters of 3% H_2_O_2_ was added to the tubes which were then placed directly on the surface of a UV transilluminator. The samples (*S*_H_ (hexane extract treated), *S*_C_ (chloroform extract treated), *S*_M_ (methanolic extract treated) and *S*_A_ (aqueous extract treated)) were irradiated for 10 min at room temperature. Two tubes without plant extract were separately maintained as controls, one non-irradiated (*C*) and one UV-irradiated (*C*_R_). *C*_R_ was irradiated similarly as the four samples. Four microliters of a tracking dye (0.25% bromophenol blue, 0.25% xylene cyanol FF and 30% glycerol) was then added to all tubes, and DNA was analyzed by gel electrophoresis on a 1% agarose horizontal slab gel in TAE buffer (pH 8). The gel was stained in ethidium bromide (1 *μ*g ml^−1^, 30 min) and photographed on Lourmat Gel Imaging System (Vilbar, France) to analyze DNA protection activity (if any) by virtue of the banding patterns.

### 2.12. Hemolysis Inhibition Assay

Assay for estimating inhibitory effect of the extracts on hemolysis was performed by the method described by Tedesco et al. [19] with some modifications. Human blood was collected in heparinized syringe and taken in tubes for the experiment. Plasma, platelets and buffy coat were removed from the blood sample by consecutive centrifugations (at 2000 rpm/224 × g for 10 min) and washings in cold PBS (pH 7.4). Aliquots of RBCs (containing 1 × 10^7^ cells) in PBS were taken in test tubes and the total volume was made up to 3 ml by addition of PBS. Extracts in diverse concentrations were added to each test tube except the control. One hundred microliters of 10 mM H_2_O_2_ was added to each tube and incubated at room temperature with continuous shaking for 2 h 30 min. Contents of the tubes were then centrifuged at 2000 rpm (224 × g) for 10 min. The supernatant thus obtained was estimated for absorbance at 540 nm. Percentage hemolysis inhibition was calculated and compared with BHT.

### 2.13. XTT Cytotoxicity Assay

Cytotoxicity of the four extracts at various concentrations was tested by the method of XTT-formazan dye formation [20]. MDA-MB-435S (human breast carcinoma) cells cultured in L-15 (Leibovitz) cell culture medium (with 10% serum) were seeded (200 *μ*l, 6 × 10^3^ cells/well) in a 96-well plate and allowed to grow for 24 h at 37°C. After incubation, medium was removed and the cells were treated with different extract concentrations prepared in 200 *μ*l L-15 medium. Extract-treated cells were re-incubated for 24 h maintaining the same conditions. After the treatment incubation period, medium containing extracts was substituted by 200 *μ*l of fresh medium followed by the addition of 50 *μ*l of XTT (0.6 mg ml^−1^ in L-15 medium) containing 25 *μ*M PMS. The plate was further incubated for 4 h in the same conditions. Absorbance was measured at 450 nm (with a 650 nm reference filter) in a Dynex Opsys MR^TM^ Microplate Reader (Dynex Technologies, VA, USA).

Percentage cytotoxicity was calculated by the following formula:

(5) $$\text{\% cytotoxicity}=\left[\frac{A_{C}-A_{T}}{A_{C}}\right]\times 100,$$

where *A*_C_ is the absorbance of the control wells and *A*_T_ is the absorbance of the test wells.

### 2.14. Statistical Analysis

All analyses were carried out in triplicates. Data were presented as mean ± standard deviation (SD). Statistical analyses were performed by one-way analysis of variance (ANOVA). Significant differences between groups were determined at *P* < .05. Experimental results were further analyzed for correlation and tested for significance by Student's *t*-test (*P* < .05). MATLAB ver. 7.0 (Natick, MA, USA), SPSS ver. 9.05 (Chicago, IL, USA) and Microsoft Excel 2007 (Roselle, IL, USA) were used for the statistical and graphical evaluations.

## 3. Results

### 3.1. DPPH Radical Scavenging Activity

**Figure 1:**
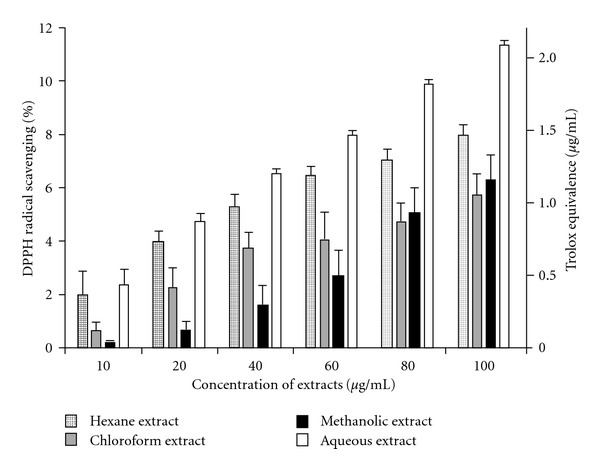
DPPH^•^ radical scavenging activity of *C. cinereum* extracts in different concentrations with Trolox equivalence (in *μ*g ml^−1^). Data expressed as mean ± SD (*n* = 3, *P* < .05).

DPPH^•^ is a stable free radical that has been extensively used to determine free radical-scavenging ability of various compounds. It has a maximum absorbance at 517 nm. Absorbance decreases when antioxidants donate protons to DPPH^•^ [11]. Quantitative analysis revealed that the aqueous extract showed the strongest DPPH^•^ radical scavenging property, followed by the hexane extract, while the chloroform and methanolic extracts lagged behind. The data are represented in Figure 1 along with the SDs and Trolox equivalence (in *μ*g ml^−1^) for the percentage scavenging.

### 3.2. ABTS Radical Scavenging Activity

**Figure 2:**
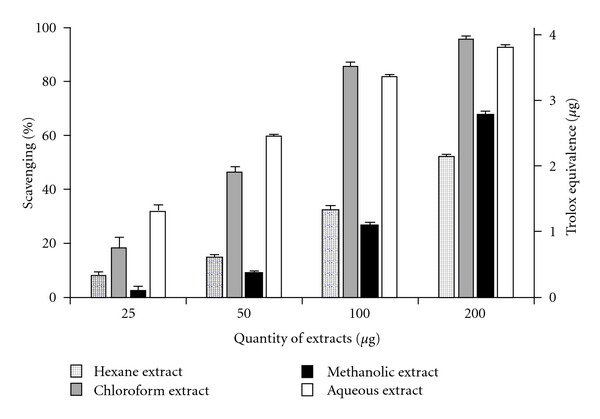
ABTS^•+^ radical scavenging activity of *C. cinereum* extracts in different concentrations with Trolox equivalence (in *μ*g). Data expressed as mean ± SD (*n* = 3, *P* < .05).

Figure 2 depicts the percentage scavenging of ABTS^•+^ radical. The relative antioxidant efficiency was compared to Trolox, and the Trolox equivalence (in *μ*g) was plotted. Although the high antioxidant property of the aqueous extract was in qualitative congruity to the results of the DPPH assay, quantitatively it was higher in the ABTS assay. Moreover, the hexane, chloroform and methanolic extracts showed significant differences (*P* < .05) in results in comparison to the DPPH assay.

### 3.3. Lipid Peroxidation Inhibition Efficiency

**Figure 3:**
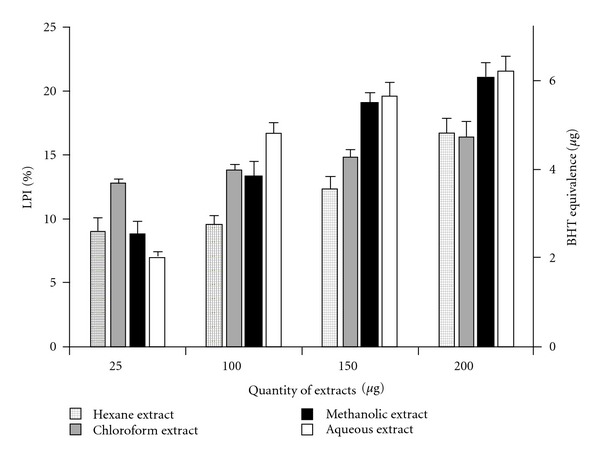
Inhibitory effect of *C. cinereum* extracts on lipid peroxidation. Percentage LPI values are given as mean ± SD (*n* = 3, *P* < .05). BHT equivalence (in *μ*g) is shown for all extract concentrations.

Intracellular and membrane lipids, when subjected to considerable oxidative stress, lose a hydrogen atom from an unsaturated fatty acyl chain, thus initiating lipid peroxidation which propagates as a chain reaction. This leads to the generation of diverse peroxides and cyclic endoperoxides which consequently form MDA. On reacting with TBA, MDA produces a pink chromogen with highest absorbance at 532 nm, thus providing an estimate of LPI [13]. A dose-dependent inhibition of lipid peroxidation was observed for all extracts (Figure 3). BHT was taken as a standard and BHT equivalence (in *μ*g) was calculated.

### 3.4. Total Phenolic Content Estimation

**Figure 4:**
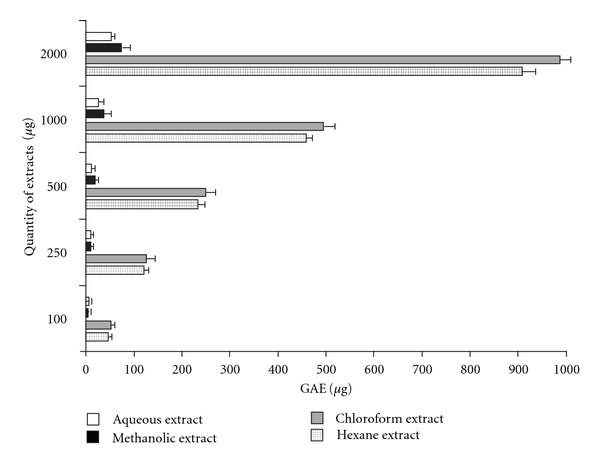
Total phenolic content in varying concentrations of *C. cinereum* extracts. Data is given in mean ± SD (*n* = 3, *P* < .05). GAE of the extracts is shown in micrograms.

Phenolic compounds have received considerable attention because of their physiological function, including antioxidant, antimutagenic and antitumor activities [21]. Variations in the quantity of total phenolics in the four extracts are presented in Figure 4. All extracts showed a concentration-dependent increase in phenolic contents. Quantitative estimation proved that the chloroform extract possessed the highest concentration of phenolics compounds among the four, closely followed by the hexane extract, while the methanolic and aqueous extracts contained very limited phenolic compounds. Total phenolic content was expressed in GAE in micrograms.

### 3.5. H_2_O_2_ Scavenging Activity

H_2_O_2_ is one of the primary free radicals in biological systems. It induces damage to the cell membrane and decreases cell viability. It also causes oxidative damage to genomic DNA and mtDNA. The protective effect of the four extracts against H_2_O_2_ was estimated. However, only the hexane extract showed considerable HPSA (Table 1), while the other three extracts did not show any significant H_2_O_2_ scavenging effect (*P* < .05).

### 3.6. Hydroxyl Radical Scavenging Activity

Hydroxyl radical ranks first among the reactive oxygen species (ROS) having the shortest half-life among all ROS. It can be produced *in vivo* in presence of both O_2_^•^^−^ radicals and transition metals via the Haber–Weiss reaction [22]. ^•^OH radical induces rigorous damage to biomolecules including proteins, lipids and DNA. Antioxidants act as inhibitors of hydroxyl radical formation and oxidative damage.

**Figure 5:**
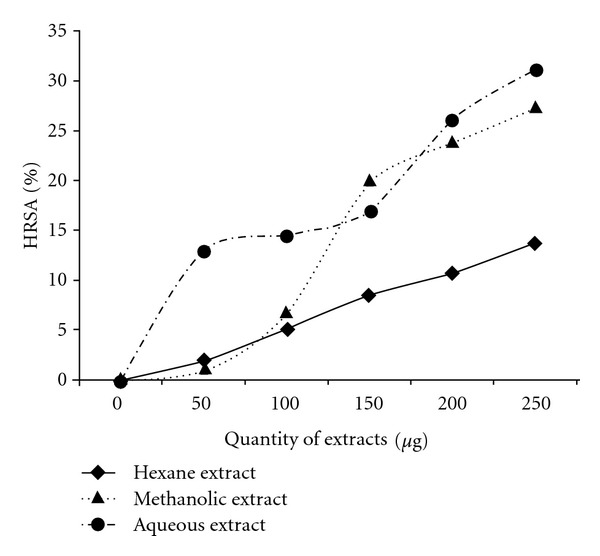
Percentage HRSA of hexane, methanolic and aqueous extracts of *C. cinereum*. Results are expressed as mean (*n* = 3, *P* < .05). Chloroform extract did not give any significant result at *P* < .05.

^•^OH radicals were generated by the ascorbate-iron-EDTA system. The ^•^OH radicals thus formed react with DMSO to yield formaldehyde, which can be detected by treatment with Nash reagent. The polar (methanolic and aqueous) extracts showed high HRSA; while the hexane extract gave lower values (Figure 5). On the other hand, the chloroform extract did not show any significant ^•^OH scavenging effect. Furthermore, significant correlation was obtained between LPI and HRSA with respect to the hexane (*r* ^2^ = 0.94), methanolic (*r* ^2^ = 0.89) and aqueous (*r* ^2^ = 0.95) extracts at *P* < .05.

### 3.7. Reducing Power Estimation

**Figure 6:**
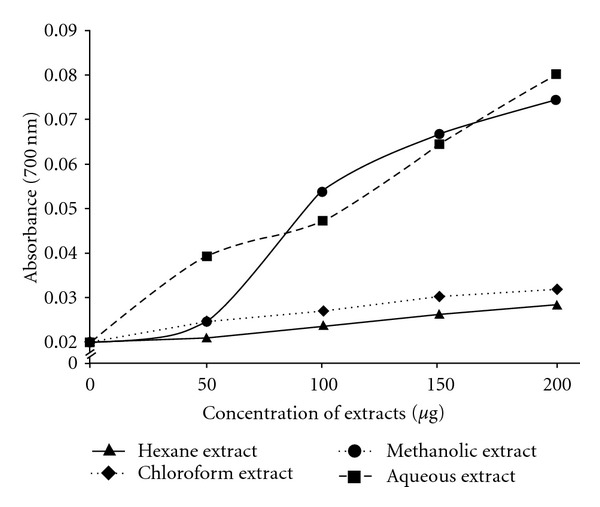
Reducing power of *C. cinereum* extracts based on measurement of Fe^+3^–Fe^+2^ transformation. Data points are mean values (*n* = 3, *P* < .05).

Antioxidant properties of compounds are associated with reducing power that is related to the presence of reductones [23], which break the free radical chain by donation of hydrogen atoms. Reducing power of the extracts was assessed by estimating the magnitude of transformation of Fe^+3^ to Fe^+2^, which is depicted by the dose-dependent increase in the absorbance at 700 nm (Figure 6). Reducing power of all extracts showed strong correlation to LPI, while only the aqueous extract's reducing power demonstrated correlation with high significance (*r* ^2^ = 0.999, *P* < .05) with HRSA.

### 3.8. DNA Damage Inhibition Efficiency

Ultraviolet-photolysis of H_2_O_2_ generates ^•^OH radicals which causes maximum oxidative damage. ^•^OH bound onto the DNA would lead to strand breakage, deoxysugar fragmentation and base modification. Moreover, oxidation of lipids induced by ^•^OH and other ROS can generate end products, such as MDA and unsaturated aldeydes, that can bind to DNA to generate mutagenic adducts [24].

**Figure 7:**
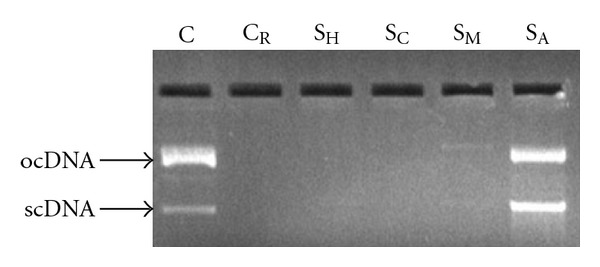
Effect of *C. cinereum* extracts (50 *μ*g) on the protection of supercoiled DNA (plasmid pBR322) against oxidative damage caused by UV-photolysis of H_2_O_2_ (3%, v/v). C = untreated non-irradiated DNA (control); *C*_R_ = untreated UV-irradiated DNA (control); *S*_H_ = UV-irradiated, hexane extract treated; *S*_C_ = UV-irradiated, chloroform extract treated; *S*_M_ = UV-irradiated, methanolic extract treated; *S*_A_ = UV-irradiated, aqueous extract treated.

Figure 7 shows the electrophoretic pattern of pBR322 DNA following UV-photolysis of H_2_O_2_ in absence (in controls *C* and *C*_R_) and presence (in samples *S*_H_, *S*_C_, *S*_M_ and *S*_A_) of the extracts. Normal pBR322 (*C*) showed two bands on agarose gel electrophoresis. The faster moving band represented the native form of supercoiled circular DNA (scDNA) and the slower moving band corresponded to the open circular form (ocDNA) [18]. UV-photolysis of H_2_O_2_ in *C*_R_, *S*_H_ and *S*_C_ caused damage to the DNA (no bands visible). *S*_A_ showed banding pattern identical to *C*, which inferred that *S*_A_ may have a high efficiency of DNA damage inhibition. *S*_M_ showed very faint bands with the same banding pattern as *C* and *S*_A_, which pointed out towards potential power of weakly inhibiting DNA damage by UV-photolysis of H_2_O_2_.

### 3.9. Hemolysis Inhibition Assay

**Figure 8:**
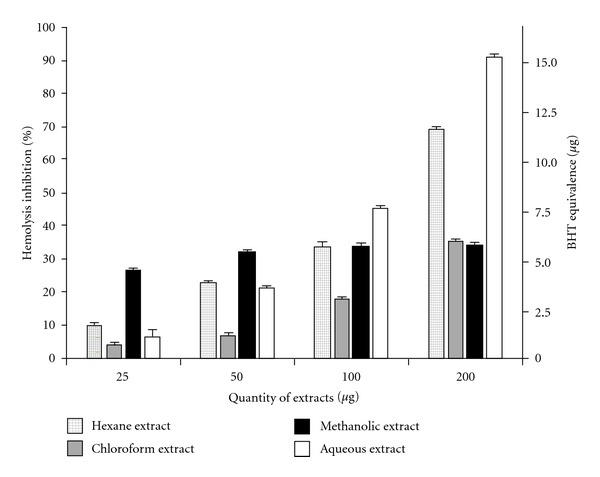
*In vitro* protective effect of *C. cinereum* extracts against H_2_O_2_-induced hemolysis in human erythrocytes. Data are expressed as mean ± SD (*n* = 3, *P* < .05).

Erythrocytes are highly susceptible to oxidative damage due to free radical attack owing to the presence of high membrane concentration of poly-unsaturated fatty acids (PUFA) and the O_2_ transport associated with redox-active hemoglobin molecules, which are potent promoters of ROS [25]. Such damage causes hemolysis of the RBCs (Figure 8).

Antioxidants have been found to protect erythrocytes from oxidative stress or increase their resistance to damage caused by oxidants [26]. All four extracts showed variable but considerable levels of dose-dependent inhibition of hemolysis by H_2_O_2_ (Figure 8). Percentage hemolysis inhibition was calculated and also expressed in BHT equivalence (in g). Hemolysis inhibition showed significant correlation (*P* < .05) with ABTS^•+^ radical scavenging activity for all extracts (*r* ^2^ (hexane) = 0.988, *r* ^2^ (chloroform) = 0.912, *r* ^2^ (methanolic) = 0.921, *r* ^2^ (aqueous) = 0.864).

### 3.10. XTT Cytotoxicity Assay

**Figure 9:**
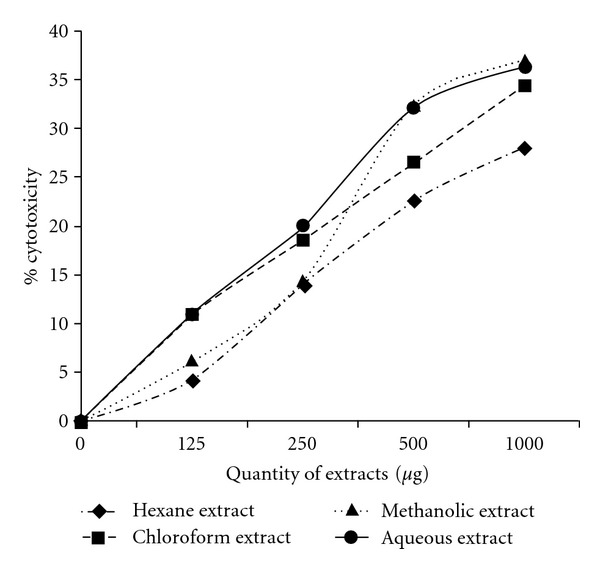
Cytotoxicity of *C. cinereum* extracts on MDA-MB-435S (human breast carcinoma) cells as estimated by XTT assay. Values given are as mean ± SD (*n* = 3, *P* < .05).

Live cells metabolically reduce XTT to a soluble product XTT-formazan, which can be estimated spectrophotometrically as a measure of cell viability [20]. MDA-MB-435S cells treated with varying concentrations of the extracts showed different levels of viability.  Figure 9 depicts the percentage cytotoxicity of the extracts. All extracts showed a dose-dependent increase in cytotoxicity, although cell viability was high even with high dosage of the extracts.

## 4. Discussion

A number of previous reports suggest therapeutic potential of *C. cinereum* against diverse types of ailments [3–10]. However, its activities against oxidative stress, cytotoxicity and genotoxicity were yet to be explored. Different free-radical generating systems were used in this study to assess the free-radical scavenging properties of the crude polar and non-polar extracts of *C. cinereum* along with evaluation of the total phenolic content, reducing power and protection against cell and DNA damage. Cytotoxicity of the extracts was also tested on MDA-MB-435S cell lines.

The observed differential scavenging activities of the extracts against DPPH^•^ and ABTS^•+^ radicals may be referred to the different mechanisms of the radical-antioxidant reactions in the two assays. The stoichiometry of reactions between the antioxidant compounds in the extracts and the DPPH^•^ and ABTS^•+^ radicals is distinctively dissimilar, which may be inferred as a reason for the difference in their scavenging potential. The diversity in radical scavenging shown in these assays may also be due to factors like stereoselectivity of the radicals or the differential solubility of the extracts in the two testing systems [27]. Therefore, a lack of any significant correlation between the two models may be justified in case of these crude extracts which contain a variety of antioxidants.

Lipid peroxidation leads to an elevated oxidative stress in cells and induces damage to genomic and mtDNA [28] which may eventually lead to unstable cytological conditions such as apoptosis or tumor generation. Peroxidation to membrane lipids (by disturbing the structural lipid bilayer) may alter the stability of ligand-binding domains on the membrane, disrupt membrane transport proteins and deactivate membrane-associated enzymes, thus eventually changing the courses and actions of the downstream signal transduction cascades [29]. Moreover, oxidative damage to membrane varies the membrane permeability and disrupts ionic channels. Hence, oxidative damage associated with lipid peroxidation may set off a great magnitude of diseases. Inhibition of lipid peroxidation is therefore a vital property of antioxidant compounds by virtue of which they can mitigate the induction and/or propagation of oxidative stress related diseases. Thus, it may be inferred that *C. cinereum* has use as a preventive medicine against oxidative stress and damage to cellular macromolecules and membranes, thus maintaining structural and functional integrity of the cells.

**Figure 10:**
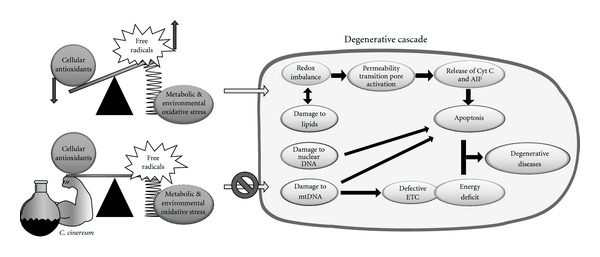
Protective effect of *C. cinereum* against degenerative diseases: Relative increase in the level of free radicals (due to metabolic and environmental oxidative stress) in cells with respect to cellular antioxidants leads to intracellular cascades that result in cellular degeneration and consequent degenerative diseases. Lower part of the figure shows the role of *C. cinereum* extracts in sustaining equilibrium between free radicals and antioxidant systems. This equilibrium inhibits oxidative damage cascade in cells, thereby mitigating degenerative diseases. AIF: apoptosis induction factors; Cyt *c*: cytochrome *c*; ETC: electron transport chain.

Experimental evidence showing considerable plasmid DNA protection by the aqueous extract against a high level of H_2_O_2_-driven oxidative damage suggests that the extract might reduce damage to mtDNA, which is particularly susceptible to oxidative damage owing to its lack of histone proteins and limited DNA-repairing enzymes. Hence, mitochondrial degenerative diseases and aging, which involve free radical generation and corresponding mtDNA and genomic DNA mutations, may find prophylaxis in the aqueous extract of *C. cinereum* (Figure 10). Although different tissue systems degenerate differentially, major sites of degeneration are those which perform extensive metabolism, such as brain, liver, kidney and muscles. These diverse systems could be made to absorb and assimilate extracts of *C. cinereum* which may play the role of an effective antioxidant defense mechanism for the tissues.

The extracts differentially showed considerable phenolic contents and high reducing power. They also showed high efficiency in inhibiting oxidative hemolysis of RBC, although showing mild levels of cytotoxicity. This cytotoxic yet protective dual character could be referred to diverse chemical properties of a variety of compounds that may be present in the crude extracts.

In conclusion, we confirmed that hexane, chloroform, methanolic and aqueous crude extracts of *C. cinereum* (whole plant) bear potent antioxidant property. Their constituents scavenge different free radicals and exert protective effects against oxidative damage to biological macromolecules like lipids and DNA. Further studies on the isolation of these compounds are in prospect. *C. cinereum* thus showed to contain considerable potential as an antithesis of free radicals, and may have prospective clinical use as a preventive medicine against various degenerative diseases and tissue aging.

## Funding

VIT University (internal funding), Vellore, India.

## Figures and Tables

**Table 1:** Percentage H_2_O_2_ scavenging activity (% HPSA) of hexane extract.

Extract concentration (in *μ*g ml^−1^)	Mean % HPSA ± SD^a^
12.5	61.07 ± 0.09
25	63.22 ± 0.87
50	65.41 ± 0.17
100	72.2 ± 0.32
200	75.44 ± 0.03

^a^Mean % HPSA ± SD at 95% confidence interval.
